# IL-27 signalling regulates glycolysis in Th1 cells to limit immunopathology during infection

**DOI:** 10.1371/journal.ppat.1008994

**Published:** 2020-10-13

**Authors:** Marcela Montes de Oca, Fabian de Labastida Rivera, Clay Winterford, Teija C. M. Frame, Susanna S. Ng, Fiona H. Amante, Chelsea L. Edwards, Luzia Bukali, Yulin Wang, Jude E. Uzonna, Rachel D. Kuns, Ping Zhang, Agnieszka Kabat, Ramon I. Klein Geltink, Edward J. Pearce, Geoffrey R. Hill, Christian R. Engwerda

**Affiliations:** 1 Immunology and Infection Laboratory, Infectious Diseases Division, QIMR Berghofer Medical Research Institute, Brisbane, Australia; 2 QIMR Berghofer Histology Facility, QIMR Berghofer Medical Research Institute, Brisbane, Australia; 3 Department of Immunology, College of Medicine, University of Manitoba, Winnipeg, Manitoba, Canada; 4 Bone Marrow Transplantation Laboratory, Cancer Division, QIMR Berghofer Medical Research Institute, Brisbane, Australia; 5 Max Plank Institute of Immunobiology and Epigenetics, Freiburg, Germany; 6 Clinical Research Division, Fred Hutchinson Cancer Research Centre, Washington, United States of America; University of Pennsylvania, UNITED STATES

## Abstract

Inflammation is critical for controlling pathogens, but also responsible for symptoms of infectious diseases. IL-27 is an important regulator of inflammation and can limit development of IFNγ-producing Tbet^+^ CD4^+^ T (Th1) cells. IL-27 is thought to do this by stimulating IL-10 production by CD4^+^ T cells, but the underlying mechanisms of these immunoregulatory pathways are not clear. Here we studied the role of IL-27 signalling in experimental visceral leishmaniasis (VL) caused by infection of C57BL/6 mice with the human pathogen *Leishmania donovani*. We found IL-27 signalling was critical for the development of IL-10-producing Th1 (Tr1) cells during infection. Furthermore, in the absence of IL-27 signalling, there was improved control of parasite growth, but accelerated splenic pathology characterised by the loss of marginal zone macrophages. Critically, we discovered that IL-27 signalling limited glycolysis in Th1 cells during infection that in turn attenuated inflammation. Furthermore, the modulation of glycolysis in the absence of IL-27 signalling restricted tissue pathology without compromising anti-parasitic immunity. Together, these findings identify a novel mechanism by which IL-27 mediates immune regulation during disease by regulating cellular metabolism.

## Author summary

Infectious diseases like visceral leishmaniasis caused by the protozoan parasites *Leishmania donovani* and *L*. *infantum* are associated with an inflammatory response generated by the host. This is needed to control parasite growth, but also contributes to the symptoms of disease. Consequently, these inflammatory responses need to be tightly regulated. Although we now recognize many of the cells and molecules involved in controlling inflammation, the underlying mechanisms mediating immune regulation are unclear. CD4^+^ T cells are critical drivers of inflammatory responses during infections and as they progress from a naïve to activated state, the metabolic pathways they use have to change to meet the new energy demands required to proliferate and produce effector molecules. In this study, we discovered that the inflammatory CD4^+^ T cells needed to control *L*. *donovani* infection switch from relying on mitochondrial oxidative pathways to glycolysis. Critically, we found the cytokine IL-27 limited glycolysis in these inflammatory CD4^+^ T cells, and in the absence of IL-27 signaling pathways, these cells expanded more rapidly to better control parasite growth, but also caused increased tissue damage in the spleen. However, pharmacological dampening of glycolysis in inflammatory CD4^+^ T cells in *L*. *donovani*-infected mice lacking IL-27 signaling pathways limited tissue damage without affecting their improved anti-parasitic activity. Together, these results demonstrate that the pathogenic activity of inflammatory CD4^+^ T cells can be modulated by altering their cellular metabolism.

## Introduction

Inflammation is a critical physiological process that mediates protection against invading pathogens. Immunoregulatory mechanisms limit inflammatory responses to prevent host tissue damage. Visceral leishmaniasis (VL) caused by *Leishmania donovani* and *L*. *infantum* is characterised by the development of a potent inflammatory response [1–3], which limits parasite growth but can also damage tissue (reviewed in [4]). A clinical feature of VL patients is hepatosplenomegaly [3, 4], which is associated with disease complications such as secondary infections, anaemia, and malnutrition, all of which can contribute to VL mortality [3–5].

Experimental VL caused by *L*. *donovani* infection in genetically susceptible mice is a useful model to study inflammation in the context of acute and chronic infection within the same host because the liver is a site of acute infection whereas chronic infection becomes established in the spleen [6,7]. Infection in the liver stimulates an inflammatory cascade including the early recruitment of innate immune cells, followed by T cells, and the rapid proliferation of the recruited lymphocytes [8]. Liver inflammation also promotes oedema, increasing the distance between parenchymal cells and blood vessels, resulting in a nutrient- and oxygen-poor microenvironment [8]. Therefore, T cells travelling from nutrient-rich secondary lymphoid tissues, such as the spleen and lymph nodes, to these sites of inflammation must adapt their metabolism to maintain their effector functions at low oxygen and nutrient levels [9]. Furthermore, the development of splenomegaly in both human and experimental VL is associated with cellular expansion and tissue reorganisation, and is likely to have consequences for cellular metabolism that are currently unclear [10,11].

We previously reported a role for Blimp-1 in inducing IL-10 production by Th1 cells to become type 1 regulatory T (Tr1) cells that protected against IFNγ- and TNF-mediated splenic pathology during infection [12]. We have also shown that CD4^+^ T cells were a major source of IL-10 that protected against tissue damage during *L*. *donovani* infection [12,13]. A link between IL-27 signalling, Tr1 cell development and IL-10 mediated control of immunopathology has been extensively reported [14–24]. However, in some instances, IL-27 can also promote inflammation by preventing regulatory T (Treg) cell expansion [25]. Hence, a better understanding about the induction and maintenance of IL-27-mediated immunoregulatory pathways is needed if they are to be exploited clinically.

IL-27p28 and Ebi3 combine to form the IL-27 heterodimer [26] that signals via the IL-27 receptor (IL-27R) composed of IL-27Rα and gp130 [27]. The engagement of IL-27 to its receptor causes phosphorylation of JAK1/2 or TYK2 and signalling via STAT1 and STAT4 to stimulate *TBET* and *IFNG* gene transcription, respectively [28,29]. Alternatively, signalling via STAT3 can induce *IL10* gene transcription [16,24,30]. A previous study reported that *Il27ra*^*-/-*^ mice developed liver damage mediated by CD4^+^ T cells and excessive IFNγ and TNF production following *L*. *donovani* infection, indicating an important role for IL-27 in protecting tissue from excess inflammation [5]. *Il27p28* and *Ebi3* mRNA levels were also reported to be significantly elevated in VL patient plasma, compared to endemic controls and CD14^+^ cells were identified as the main source of IL-27 [15].

In addition to protecting against tissue damage, IL-27 was postulated to promote susceptibility to VL by suppressing IL-17-mediated neutrophil recruitment. *Ebi3*^*-/-*^ mice had reduced IFNγ levels, compared to wild type controls, but lower parasite burdens associated with exacerbated IL-17A production leading to a greater neutrophil influx [31]. In humans, the severity of VL caused by *L*. *infantum* positively correlated with serum IL-6, IL-27 and sCD14 levels [32]. Similarly, there was a strong association between serum IFNγ, IL-27, IL-10, IL-6 and sCD14 levels and the degree of hepatosplenomegaly, neutropenia and thrombocytopenia in VL patients infected with *L*. *infantum* [32]. However, despite these reports, the cellular and molecular mechanisms underpinning IL-27 functions during VL remains poorly understood.

Cellular metabolism is tightly linked with immune cell function. Nutrient and oxygen availability, activation status, tissue site and transcriptional programming combine to orchestrate a functional immune response. Naïve CD4^+^ T cells favour energy production over biosynthesis and generally rely on mitochondrial oxidative pathways, fuelled by fatty acid or amino acid oxidation [33]. Differentiated Th1 cells rely on glycolysis and glutaminolysis to support their growth and proliferation, where α-ketoglutarate has been postulated to act as a metabolic regulator by promoting *Tbet* expression and mTORC1 signalling [34,35]. Glucose uptake and aerobic glycolysis is essential for IFNγ production by Th1 cells [36,37]. Metabolic reprogramming to glycolysis accommodates for an increase in biomass to cope with the new energy demands of the cell, but also allows for the generation of metabolic intermediates that feed into other biosynthetic pathways, such as the pentose phosphate pathway or TCA cycle. However, the mechanisms controlling these changes in CD4^+^ T cell metabolism remain poorly defined.

Here, we examined the role of IL-27 signalling in regulating CD4^+^ T cell differentiation and balancing anti-parasitic immunity and tissue pathology in experimental VL caused by *L*. *donovani* infection of C57BL/6 mice. We identified IL-27 as an important regulator of glycolysis in Th1 cells that limited host tissue damage in the spleen following infection. Importantly, we demonstrate that modulation of glycolysis during infection has therapeutic potential to reduce tissue damage without compromising control of parasite growth.

## Results

### IL-27 signalling impedes control of parasite growth and determines the balance between Th1 and Tr1 cells during infection

**Fig 1:**
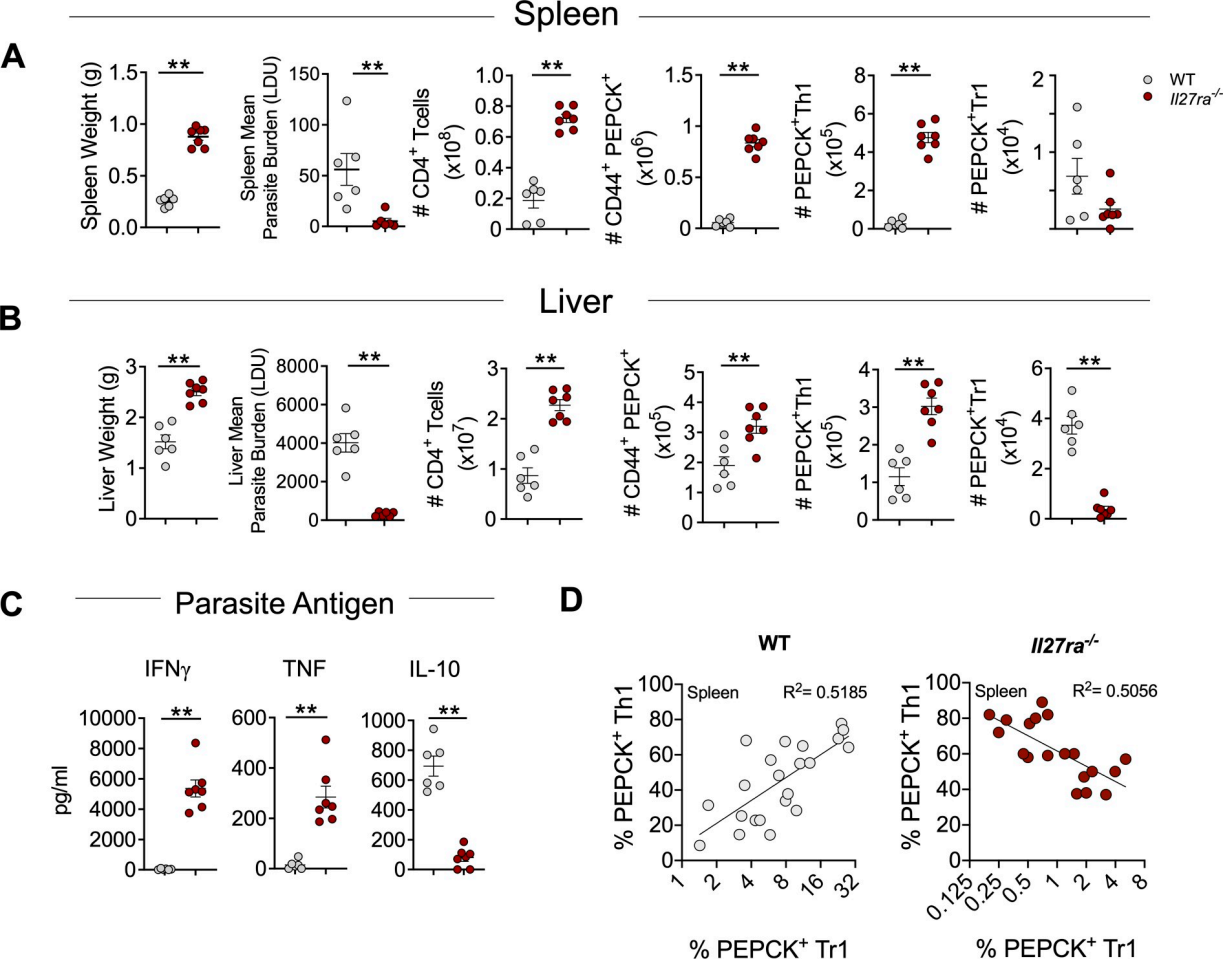
IL-27 signalling impedes control of parasite growth and determines the balance between Th1 and Tr1 cells during infection. WT and *Il27ra*^*-/-*^ mice were infected with 2x10^7^ *L*. *donovani* amastigotes i.v.. Organ pathology, parasite burdens and cellular immune responses were measured in both the spleen (**A**) and liver 14 days p.i. (**B**). (**A**) Left to right: spleen weights shown in grams (g). Parasite burdens in the spleen were expressed by Leishman Donovan Units (LDU) where organ weight is multiplied by the number of parasites per 1000 nuclei in each organ. CD4^+^ T cell numbers were measured by flow cytometry as CD4^+^ TCRβ^+^. Antigen specific CD4^+^ T cell numbers defined as CD44^+^ PEPCK^+^. Antigen specific Th1 cell numbers defined as CD44^+^ PEPCK^+^ Tbet^+^ IFNγ^+^. Antigen specific Tr1 cell numbers defined as CD44^+^ PEPCK^+^ IL-10^+^ IFNγ^+^. (**B**) Left to right: liver weights shown in grams (g). Parasite burdens in the liver were expressed by Leishman Donovan Units (LDU). CD4^+^ T cell numbers are shown. Antigen specific CD4^+^ T cell numbers are shown. Antigen specific Th1 cell numbers are shown. Antigen specific Tr1 cell numbers are shown. (**C**) Splenocytes were cultured with or without the presence of *L*. *donovani* antigen and cytokines in culture supernatants were measured by CBA, 72 hours post re-stimulation. (**D**) WT (n = 22) and *Il27ra*^*-/-*^ (n = 19); Correlation of antigen-specific Th1 cells and antigen-specific Tr1 cells in the spleens of WT and *Il27ra*^*-/-*^ mice at day 14 p.i. shown by linear regression analysis. Data shown is representative of 3 independent experiments performed with n = 6–7 mice per group, in each experiment and are presented as mean ± SEM, **p<0.01, Mann-Whitney U test.

To better understand the role of IL-27 signalling in the development of anti-parasitic immunity during VL, we infected *Il27ra*-deficient C57BL/6 (*Il27ra*^*-/-*^) and wild-type (WT) C57BL/6 control mice with *L*. *donovani* and examined disease outcomes. Consistent with previous findings [5], *Il27ra*^*-/-*^ mice had significantly increased spleen and liver weights 14 days post infection (p.i.), compared to WT controls, indicating early development of tissue pathology (Fig 1A and 1B). However, we also noted reduced parasite burdens in the spleen and liver, associated with increased numbers of antigen specific CD4^+^ T cells and Th1 cells, relative to WT controls (Fig 1A and 1B, S1A–S1C Fig), as well as elevated levels of IFNγ and TNF in the serum of infected *Il27ra*^*-/-*^ mice, compared to WT controls (S1D Fig). Antigen-specific CD4^+^ T cells were measured using the PEPCK_335-351_ tetramer comprising a parasite peptide found in all pathogenic *Leishmania* species [38] (S1A and S1B Fig). Interestingly, we found reduced numbers of PEPCK^+^ Tr1 cells in the liver (Fig 1B), and reduced frequencies of Tr1 cells in both spleen and liver *Il27ra*^*-/-*^ mice, compared to WT mice (S1C Fig). This was accompanied by an increased frequency of polyclonal Th1 cells in *L*. *donovani*-infected *Il27ra*^*-/-*^ mice compared to infected WT mice (S1F Fig). In addition, IFNγ and TNF levels were elevated, and IL-10 levels were reduced following stimulation of splenocytes from *Il27ra*^*-/-*^ mice relative to WT mice with parasite antigen at day 14 p.i. (Fig 1C). Therefore, we hypothesised that the more rapid development of splenomegaly in *L*. *donovani*-infected *Il27ra*^*-/-*^ mice was caused by an unbalanced Th1 and Tr1 cell response. This was supported by an inverse correlation between antigen-specific Th1 and Tr1 cell frequency in *Il27ra*^*-/-*^ mice in the spleen 14 days p.i., as opposed to the positive correlation apparent in WT controls (Fig 1D).

To establish whether IL-27 signalled directly to macrophages to affect their ability to control parasite growth, we isolated peritoneal macrophages from *Il27ra*^*-/-*^ mice and WT controls, infected them and then cultured them with or without IFNγ. We found no impact of *Il27ra*-deficiency on the establishment of infection or growth of parasites in host macrophages (S1G Fig). Furthermore, the addition of IFNγ resulted in improved control of parasite growth in both *Il27ra*^*-/-*^ and WT macrophages, while the addition of IL-27 had no significant impact on the establishment of infection or growth of parasites in WT macrophages (S1G Fig). Together, these data indicate that the increased hepatosplenomegaly associated with *L*. *donovani* infection in *Il27ra*^*-/-*^ mice relative to WT controls was not related to direct IL-27 signalling to infected macrophages, but instead was strongly associated with changes in the balance between Th1 and Tr1 cells.

### IL-27 signalling protects splenic tissue against IFNγ- and TNF-mediated pathology during infection

**Fig 2:**
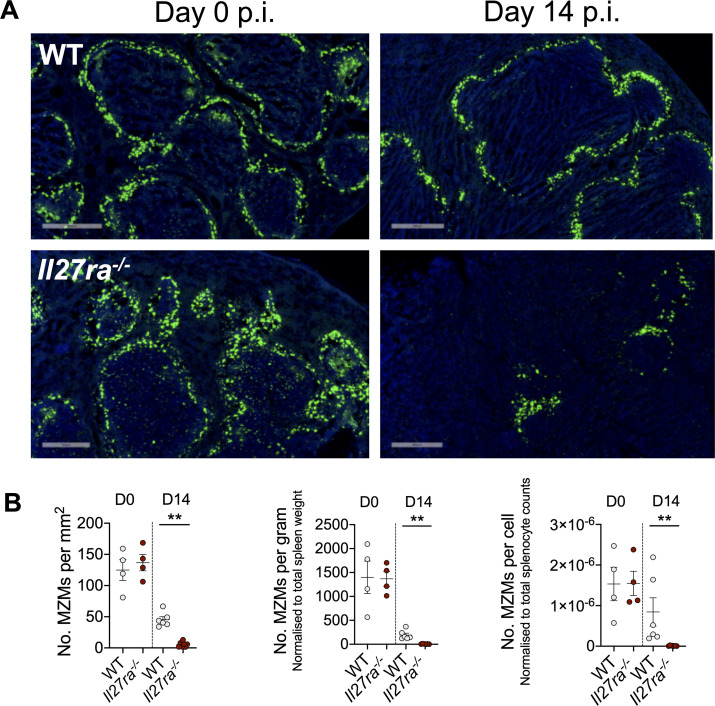
IL-27 signalling protects splenic architecture against TNF-mediated pathology during infection. (**A**) WT and *Il27ra*^*-/-*^ mice were infected with 2x10^7^ *L*. *donovani* amastigotes i.v.. Mice were injected with 100μg of FITC dextran (in saline) i.v. and euthanized 2 hours later. Spleens harvested on day 0 and day 14 p.i.. (**B**) Marginal zone macrophages were quantified in 20μM thick sections using Metamorph software as total number of marginal zone macrophages (MZMs) per millimetre squared, normalised to total spleen weight and to total splenocyte counts. Scale bar: 500μM. Data shown is representative of 2 independent experiments performed with n = 4–7 mice per group, in each experiment and are presented as mean ± SEM, **p<0.01, Mann-Whitney U test.

*L*. *donovani* infected *Il27ra*^*-/-*^ mice have previously been shown to develop CD4^+^ T cell-mediated liver pathology [5]. During VL, splenomegaly is characterised by white pulp hyperplasia and IFNγ- and TNF-mediated killing of marginal zone macrophages, resulting in dysregulated lymphocyte trafficking [39]. Similar to our previous findings in *L*. *donovani*-infected mice with impaired development of Tr1 cells [12], *Il27ra*^*-/-*^ mice exhibited a significant loss of marginal zone macrophages (MZM) 14 days p.i., compared to infected WT mice (Fig 2A and 2B). The decrease in MZM numbers was not simply a result of tissue or cellular expansion caused by splenomegaly in *Il27ra*^*-/-*^ mice because when MZM numbers were normalised to spleen weight or cell number, the same pattern of MZM loss was observed (Fig 2B). As mentioned above, a likely explanation for the increased splenic pathology observed in *Il27ra*^*-/-*^ mice was the increased ratio of Th1:Tr1 cells in these mice following *L*. *donovani* infection, compared to WT animals (Fig 1D), although cytokine blocking studies in *Il27ra*^*-/-*^ mice are needed to confirm this.

### IL-27 signalling mediates mitochondrial changes in the spleen but not liver during infection

**Fig 3:**
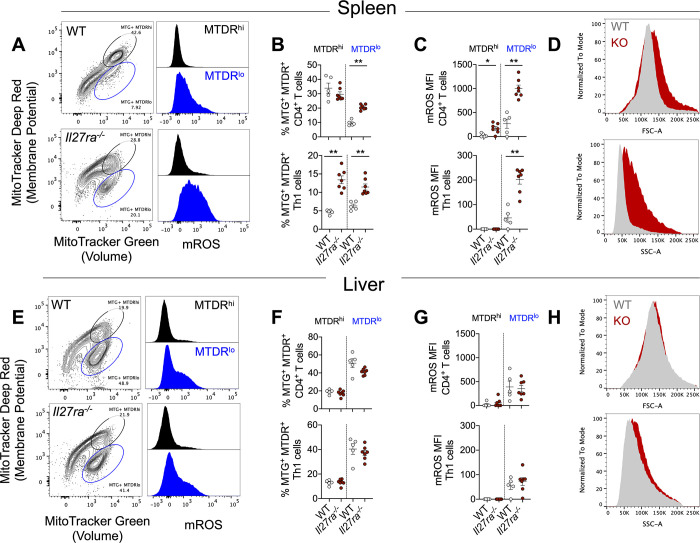
IL-27 signalling mediates mitochondrial changes in the spleen but not the liver during infection. (**A, E**) WT and *Il27ra*^*-/-*^ mice were infected with 2x10^7^ *L*. *donovani* amastigotes i.v.. Spleen and liver cells were harvested and stained for CD4, TCRβ, CXCR3 and CXCR5 along with MitoTracker Green (volume), MitoTracker DeepRed (membrane potential) and MitoSOX (mitochondrial ROS) to assess mitochondria in CD4^+^ T cells by flow cytometry. All plots are gated on CD4^+^ T cells. MTDR^hi^ (black gate and histogram), MTDR^lo^ (blue gate and histogram). CD4^+^ T cells defined as CD4^+^ TCRβ^+^ and Th1 cells defined as CD4^+^ TCRβ^+^ CXCR3^+^ CXCR5^-^ (**B, F**) Mitochondrial mass and membrane potential are shown as MitoTracker Green^+^ MitoTracker DeepRed^+^ on CD4^+^ T cells and Th1 cells at day 14 p.i.. (**C, G**) Mitochondrial Reactive Oxygen Species (mROS) measured by flow cytometry on CD4^+^ T cells and Th1 cells at day 14 p.i.. (**D, H**) Forward (FSC-A) and size scatter (SSC-A) histogram plots, WT (grey) and Il27ra^-/-^ (red). Data shown is representative of 2 independent experiments performed with n = 5–7 mice per group, in each experiment and are presented as mean ± SEM, **p<0.01, *p<0.05, Mann-Whitney U test.

Cellular metabolism is critical in shaping immune responses during infection. Naïve T cells predominantly use oxidative phosphorylation and once activated upregulate glycolysis to cope with the energy demands of newly activated cells [40]. During *L*. *donovani* infection, chronic infection becomes established in the spleen, whereas the liver is a site of self-resolving infection mediated by a Th1 cell-dominated granulomatous response [41–43]. To explore whether IL-27 signalling was associated with changes in CD4^+^ T cell metabolism during infection, we first measured mitochondrial mass and membrane potential using MitoTracker dyes in WT and *Il27ra*^*-/-*^ CD4^+^ T cells and Th1 cells 14 days after *L*. *donovani* infection. Here, we defined Th1 cells as CD4^+^ TCRβ^+^ CXCR3^+^ CXCR5^-^ as surrogate surface markers due to the sensitivity of MitoTracker dyes to fixation and permeabilization methods (S1E Fig). We found that *Il27ra*^*-/-*^ CD4^+^ T cells and Th1 cells had a two-fold increase in the MitoTracker Green^+^ MitoTracker Deep Red^lo^ population in the spleen (Fig 3A and 3B), but not the liver (Fig 3E and 3F), compared to the same cell populations from WT mice. Interestingly, this MTG^+^ MTDR^lo^ population in the spleen of *Il27ra*–deficient mice exhibited a dramatic increase in mROS expression measured by MitoSOX Red (Fig 3A–3C), indicative of highly activated CD4^+^ T cells [44,45]. Therefore, we hypothesised that the increased ROS production in *Il27ra*^*-/-*^ CD4^+^ T cells, and in particular Th1 cells, was due to the increased activation status of these cells, relative to WT cells. In support of this idea, we found CD4^+^ T cells from the spleens of *Il27ra*^*-/-*^ mice were larger in size and granularity, compared to the same cells from WT controls (Fig 3D), and again these differences were not found in the liver (Fig 3G and 3H). Collectively, these data indicate that IL-27 signalling suppressed mitochondrial changes in splenic CD4^+^ T cells during *L*. *donovani* infection that limit their activation status, potentially contributing to the immunosuppression observed in this tissue [46]. Furthermore, these mitochondrial changes observed in splenic CD4^+^ T cells were not simply caused by an increase in Th1 cell frequencies in *Il27ra*^*-/-*^ mice because we also observed these changes in MTG^+^ MTDR^+^ Th1 cells between WT and *Il27ra*^*-/-*^ mice. The minimal differences observed between WT and *II27ra*^*-/-*^ CD4^+^ T cells and Th1 cells in the liver reflects the improved control of parasite load in this tissue, compared to the spleen [11, 43, 46]. We hypothesised that at day 14 p.i. when parasite load begins to resolve in the liver, but not the spleen, the activation and metabolic potential of hepatic CD4^+^ T cells had already reached their maximum, hence no further increase in mROS or cell size and granularity was observed.

### Th1 cells are more glycolytic than Tr1 cells in vivo

**Fig 4:**
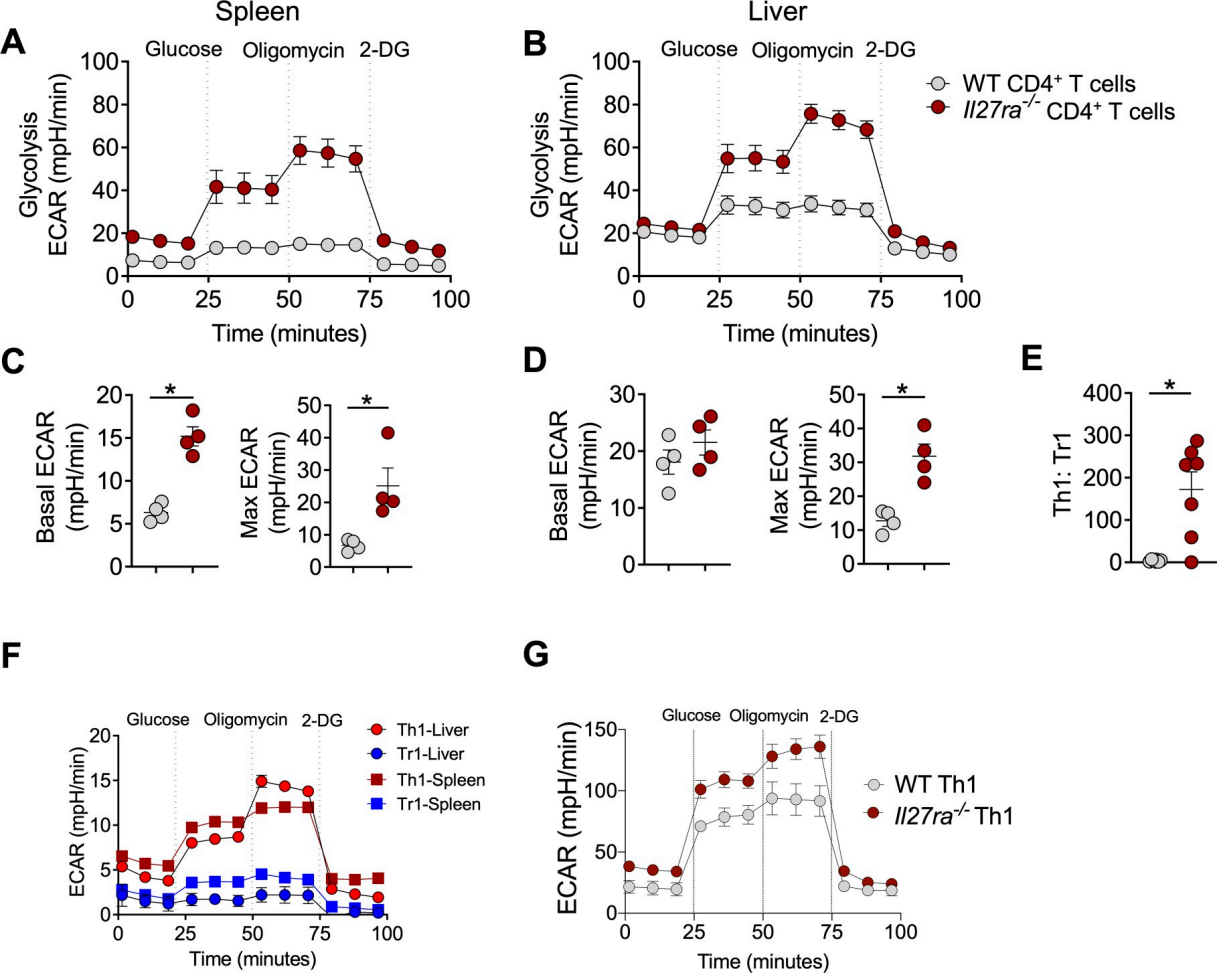
Th1 cells are more glycolytic than Tr1 cells in vivo during infection. WT and *Il27ra*^*-/-*^ mice were infected with 2x10^7^ *L*. *donovani* amastigotes i.v.. Splenic and hepatic CD4^+^ T cells were MACS purified and assayed on the Seahorse XF96 using the glycolysis stress test kit at day 14 p.i. *ex vivo*. Total extracellular acidification rate (ECAR) was assessed after the addition of glucose, oligomycin and 2-DG at indicated times in the (**A**) spleen and (**B**) liver. Basal ECAR and maximal glycolysis (final measurement after the last oligomycin injection–final measurement after the last glucose injection) measured in the (**C**) spleen and (**D**) liver. (**E**) Th1:Tr1 cell ratio determined by flow cytometry. (**F**) *Il10gfp x Ifngyfp x foxp3rfp* mice were infected with 2x10^7^ *L*. *donovani* amastigotes i.v.. Splenic and hepatic CD4^+^ T cells were sorted on the ARIA III for Th1 (IFNγ^+^ IL-10^-^) and Tr1 (IFNγ^+^ IL-10^+^) cells 14 days p.i.. Total extracellular acidification rate (ECAR) was assessed after the addition of glucose, oligomycin and 2-DG at indicated times in the spleen and liver ex vivo. (**G**) Splenic CD4^+^ T cells MACS purified from uninfected WT and *Il27ra*^*-/-*^ mice and polarised under Th1 conditions for 72 hours and then assayed on the Seahorse XF96. Glycolysis (ECAR) measured in all conditions. Data shown is representative of 2 independent experiments performed with n = 3–7 mice per group, in each experiment and are presented as mean ± SEM, *p<0.05, Mann-Whitney U test.

We next investigated how IL-27 signalling affects mitochondrial respiration in response to glucose. In CD4^+^ T cells from the spleen, we measured improved mitochondrial respiration (oxygen consumption rate, OCR) in response to glucose, in the absence of IL-27 signalling (S2A Fig), whereas minimal differences in OCR between WT and *Il27ra*^*-/-*^ CD4^+^ T cells were found in the liver (S2B Fig), supporting the above findings of tissue-specific differences in the metabolic status of CD4^+^ T cells, where it appears that hepatic CD4^+^ T cells reached their peak metabolic state earlier than splenic CD4^+^ T cells. However, we found increased aerobic glycolysis (extracellular acidification rate, ECAR) in the absence of IL-27 signalling in CD4^+^ T cells from both spleen (Fig 4A) and liver (Fig 4B). Interestingly, *Il27ra*^*-/-*^ CD4^+^ T cells from the spleen were significantly more glycolytic than their WT counterparts, even at baseline (Fig 4C). Basal ECAR (absence of glucose) between WT and *Il27ra*^*-/-*^ CD4^+^ T cells in the liver was not different, and only when glucose became available in saturated amounts, did we observe a difference in glycolysis between WT and *Il27ra*^*-/-*^ CD4^+^ T cells (Fig 4D). Thus, despite differences in glucose utilisation between WT and *Il27ra*^*-/-*^ CD4^+^ T cells in the liver and spleen at baseline, the addition of a saturating amount of glucose resulted in the rapid breakdown of glucose into pyruvate, measured by the maximum ECAR (Fig 4C and 4D). The number of live WT and *Il27ra*^*-/-*^ CD4^+^ T cells recovered and assayed was similar (~80%), and therefore did not account for the above differences at baseline. However, we cannot say whether these cells were more or less susceptible to cell death during the assay itself. Furthermore, once this glycolytic pathway was activated, *Il27ra*^*-/-*^ CD4^+^ T cells were significantly more glycolytic compared to WT cells in both tissues, as indicated by the max ECAR readings (Fig 4C and 4D). Because we measured aerobic glycolysis on bulk CD4^+^ T cell populations from WT and *Il27ra*^*-/-*^ mice, it is important to note that *Il27ra*^*-/-*^ mice exhibit a significantly higher Th1:Tr1 ratio (Fig 4E). Hence, differences in the capacity for aerobic glycolysis between Th1 and Tr1 cells may help to explain the increased ECAR in *Il27ra*^*-/-*^ CD4^+^ T cells, particularly in the liver (Fig 4B). Indeed, we found Th1 cells were more glycolytic than Tr1 cells *in vivo* 14 days p.i. (Fig 4F), providing functional support for our explanation of IL-27-mediated metabolic changes above. In addition, Th1 cells polarised from *Il27ra*^*-/-*^ CD4^+^ T cells were more glycolytic compared to WT Th1 cells *in vitro* (Fig 4G and S2C Fig). Previous studies reported IL-10 mediated inhibition of glycolysis in macrophages [47] and CD4^+^ T cells have been shown to respond to IL-10 signalling during *L*. *donovani* infection [13]. Since *Il27ra*^*-/-*^ CD4^+^ T cells displayed impaired IL-10 production, we examined whether this could account for the increase in glycolysis we observed. We found that the glycolytic capacity of CD4^+^ T cells from *Il27ra*^*-/-*^ mice was partially reduced upon the addition of IL-10 (S2D Fig). However, this did not reach statistical significance suggesting that IL-10 indirectly or only partially regulates glycolysis in CD4^+^ T cells in the absence of IL-27 signalling during *L*. *donovani* infection. Collectively, these data support previous work showing Th1 cells were more glycolytic than their regulatory counterparts [48], and importantly demonstrate for the first time that IL-27 signalling is an important regulator of glycolysis in Th1 cells.

### IL-27 signalling limits glycolysis in Th1 cells to restrict tissue pathology

**Fig 5:**
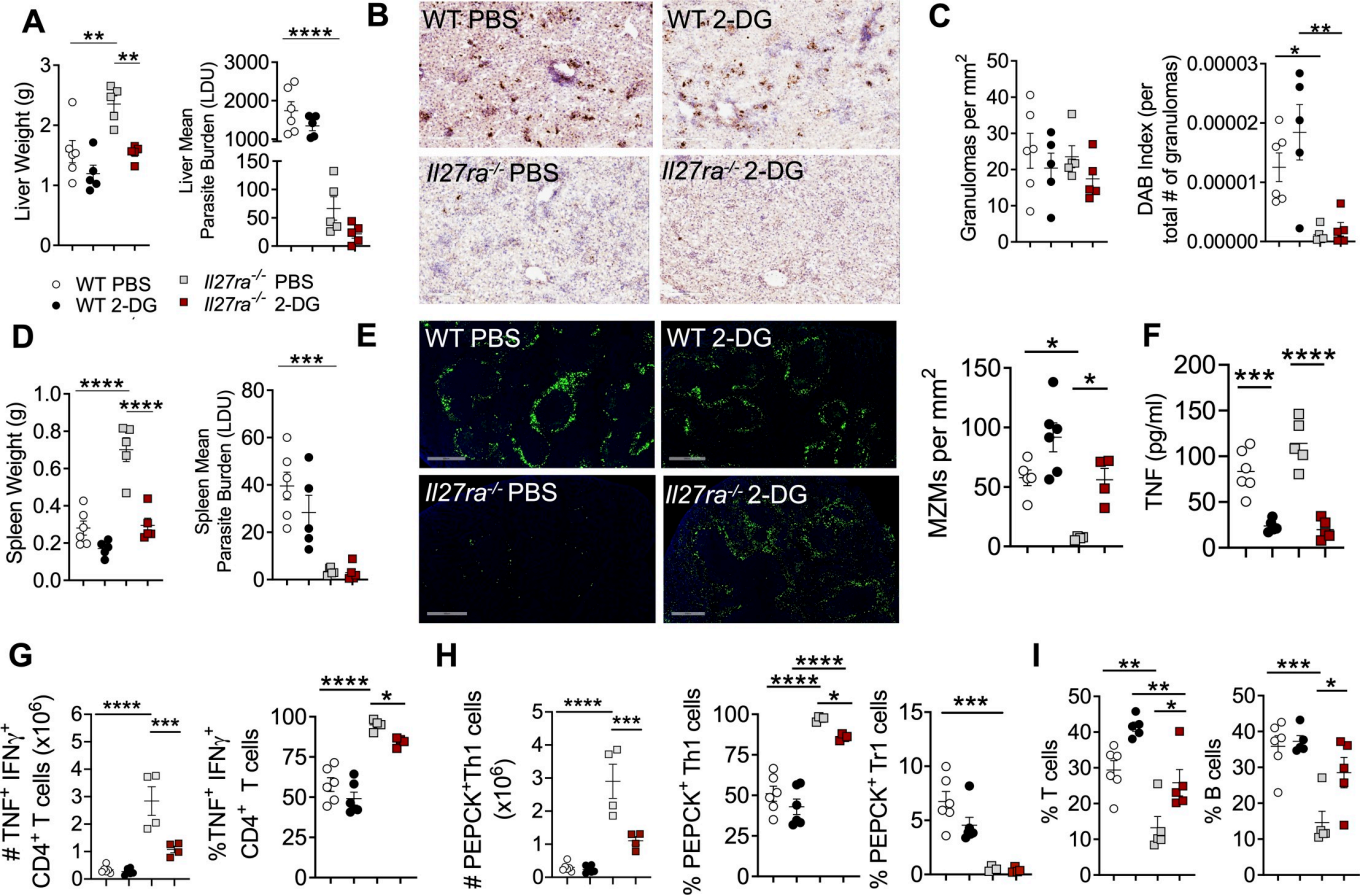
IL-27 signalling limits glycolysis in Th1 cells and protects against tissue pathology during infection. WT and *Il27ra*^*-/-*^ mice infected with 2x10^7^ *L*. *donovani* amastigotes i.v.. Mice were treated with PBS (controls) or 1g/kg of 2-DG daily i.p. beginning at day 7 p.i., until day 14 p.i. Organs were harvested 14 days p.i. and processed for cellular analysis. (**A**) Liver weights (g) and parasite burdens measured in Leishman Donovan Units (LDU: number of amastigotes per 1000 nuclei x organ weight) 14 days p.i.. (**B**) Liver sections (scale bars: 200μM) imaged on the Aperio XT Turbo slide scanner. (**C**) Total granulomas and DAB quantification (DAB index) were performed using Image J and adjusted to the section area in mm^2^. (**D**) Spleen weights (g) and parasite burdens measured in LDU 14 days p.i.. (**E**) Marginal zone macrophages (MZMs) were quantified in the spleen by injecting mice with 100μg of FITC dextran i.v. (scale bars: 500μΜ) and imaged of the Aperio FL slide scanner. MZMs were quantified using Metamorph per mm^2^. (**F**) TNF levels (pg/mL) measured in the serum 14 days p.i. (**G**) Number and frequencies of splenic CD4^+^ T cells co-producing IFNγ and TNF was measured by flow cytometry 14 days p.i. (**H**) Number and frequencies of antigen-specific Th1 and Tr1 cells was measured by flow cytometry 14 days p.i. (**I**) T (TCRβ^+^) and B (B220^+^ CD19^+^) cell frequencies measured by flow cytometry 14 days p.i. Data shown is representative of 3 independent experiments performed with n = 4–6 mice per group, in each experiment and are presented as mean ± SEM, ****p<0.0001, ***p<0.0005, **p<0.005, *p<0.05, One-Way ANOVA with Tukey’s multiple comparisons test.

To explore the effects of increased glycolysis in Th1 cells in the absence of IL-27 signalling on disease outcomes, we infected WT and *Il27ra*^*-/-*^ mice with *L*. *donovani* and treated with either 2-DG (inhibitor of glycolysis) or PBS vehicle beginning at day 7 p.i., every day until day 14 p.i.. 2-DG treatment reduced liver weights with limited impact on parasite burdens in *Il27ra*^*-/-*^ mice (Fig 5A). It has previously been shown that large granulomas with diffuse foci of inflammatory infiltrates form in the liver in the absence of IL-27 signalling during *L*. *donovani* infection [5]. However, we found no difference in the total number of granulomas in WT and *Il27ra*^*-/-*^ mice at day 14 p.i. (Fig 5B and 5C), but a significant reduction in parasites (indicated by DAB staining) within the granulomas of *Il27ra*^*-/-*^ mice, compared to WT controls (Fig 5C), consistent with earlier parasite counts in tissue impression smears (Fig 5A). Importantly, 2-DG treatment resulted in a reduction in gross splenic pathology in *Il27ra*^*-/-*^ mice without impairing parasite control (Fig 5D), suggesting that increased glycolysis in the absence of IL-27 signalling may be targeted to reduce infection-induced inflammation, without impacting anti-parasitic immunity. Critically, the splenic architecture was preserved in the *Il27ra*^*-/-*^ mice treated with 2-DG (Fig 5E), and this was associated with reduced TNF levels in the serum (Fig 5F) and TNF production by splenic CD4^+^ T cells 14 days p.i. (Fig 5G), relative to PBS-treated *Il27ra*^*-/-*^ mice. In addition, we measured reduced numbers and frequencies of Th1 cells, but no effect on Tr1 cells (Fig 5H). Moreover, 2-DG had no effect on the frequencies of conventional or antigen-specific IL-10 producing Treg cells (S3A Fig). Together, these results suggest that 2-DG treatment limited Th1 cell development and function in the absence of IL-27 signalling to a level that prevented tissue damage but allowed control of parasite growth. The effects of 2-DG are not T cell specific and therefore we measured other lymphocyte and myeloid populations by flow cytometry and found no obvious differences (S3B and S3C Fig). CD4^+^ T cells were the predominant source of IFNγ in all groups (S3D Fig). Collectively, these data suggest a primary effect of 2-DG in *Il27ra*^*-/-*^ mice on IFNγ production, and in particular on the development of Th1 cells, compared to other cell types assessed. We previously reported that IFNγ signalling was critical for induction of TNF-mediated splenic pathology and found improved lymphocyte trafficking as a result of preserved splenic architecture following TNF blockade in *L*. *donovani*-infected mice [12]. Indeed, the preservation of splenic architecture in the *Il27ra*^*-/-*^ mice treated with 2-DG resulted in increased T and B cell frequencies in this organ (Fig 5I). Taken together, these results indicate that IL-27 signalling limits glycolysis in Th1 cells *in vivo* in order to protect tissue architecture against infection-induced inflammation.

### Th1 cells rely on metabolic intermediates generated during glycolysis to produce pro- inflammatory cytokines

**Fig 6:**
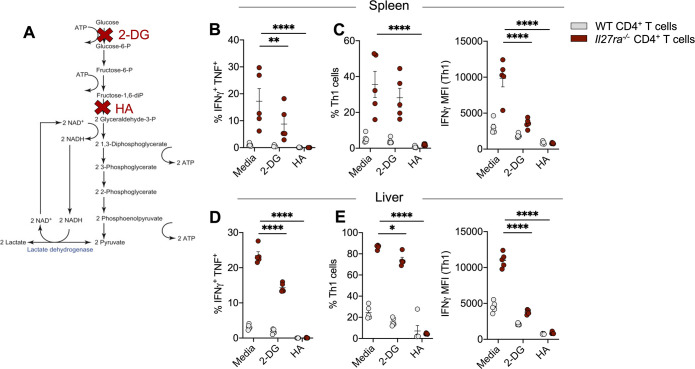
Th1 cells rely on metabolic intermediates generated during glycolysis to produce pro-inflammatory cytokines. (**A**) 2x10^5^ CD4^+^ T cells were MACS purified from the spleens and livers of *L*. *donovani* infected WT and *Il27ra*^*-/-*^ mice at day 14 p.i. and treated with either media, or 1mM of either 2-DG or heptelidic acid (HA) for 1 hour and re-stimulated with PMA/Ionomycin in the presence of monensin for 3 hours. Red crosses show the targets of 2-DG and HA within the glycolysis pathway. (**B**) Frequencies of IFNγ^+^ TNF^+^ CD4^+^ T cells measured in the spleen by flow cytometry. (**C**) IFNγ^+^ Τbet^+^ (Th1) cell frequencies measured in the spleen and IFNγ expression shown as the Mean Fluorescence Intensity (MFI). (**D**) Frequencies of IFNγ^+^ TNF^+^ CD4^+^ T cells measured in the liver by flow cytometry. (**E**) IFNγ^+^ Τbet^+^ (Th1) cell frequencies measured in the liver and IFNγ expression shown as the Mean Fluorescence Intensity (MFI). Data shown is representative of 2 independent experiments performed with n = 5 mice per group, in each experiment and are presented as mean ± SEM, ****p<0.0001, **p<0.005, *p<0.05, Two-Way ANOVA with Sidak’s multiple comparisons test.

Given 2-DG targets hexokinase at the beginning of the glycolysis pathway, we next sought to determine whether the reduced IFNγ and TNF production was a direct result of blockade of the glycolysis pathway itself or whether the administration of 2-DG was limiting the availability of metabolic intermediates for other metabolic pathways required by CD4^+^ T cells [49]. In addition to 2-DG, we used heptelidic acid (HA) which inhibits GAPDH (Fig 6A). Interestingly, splenic Th1 cell and IFNγ^+^ ΤNF^+^ CD4^+^ T cell frequencies were reduced in the 2-DG and HA treated groups (Fig 6B and 6C, S4A Fig). The same effect was also observed in the liver (Fig 6D and 6E). Collectively, these data show that IL-27 signalling limits glycolysis in Th1 cells and their ability to produce IFNγ and TNF is impaired when glycolysis and the metabolic intermediates generated from glycolysis are inhibited. Importantly, this reduction in cytokine production was directed towards Th1 cells, as we found no changes in Tr1 cell frequencies (S4B Fig).

## Discussion

We previously showed that Blimp1-mediated IL-10 production by CD4^+^ T cells protects against tissue damage in the spleen following *L*. *donovani* infection [12]. Here we show that IL-27 signalling plays a pivotal role in generating Tr1 cells during infection, whereby *Il27ra*^*-/-*^ mice had improved parasite control but accelerated splenic pathology, associated with an increased proportion of Th1 cells, relative to Tr1 cells. These findings along with others [14,20,50] highlight the importance of IL-27 signalling in regulating T cell responses during infection to protect host tissue. Here we show early MZM loss in *Il27ra*^*-/-*^ mice suggesting IL-27 signalling protects against IFNγ- and TNF-mediated pathology. IL-27 can exert its anti-inflammatory effects via suppression of IL-17 [14,23,31,51,52] and stimulation of IL-10 production [14,15,23,24,53–55]. These latter studies support our findings of reduced IL-10 production and Tr1 cell frequencies in the *Il27ra*^*-/-*^ mice.

Th1 cells are important for controlling many intracellular pathogens [56,57]. However, excess inflammation contributed by these cells can damage tissue and promote dysfunctional immune responses (reviewed in [19]). IL-27 signalling and its role in limiting Th1 cell-mediated inflammation has been reported in many experimental settings, including, encephalomyelitis [58], inflammatory bowel disease [59], tuberculosis [60], trypanosomiasis [61], malaria [20] and leishmaniasis [5]. IL-27 signalling is thought to limit the formation of pathogenic Th1 cells by suppressing CCR5 [62] and IL-2 [63,64] expression. Although IL-12-mediated IL-10 induction contributes to these effects [12,65], the mechanism underpinning this immunoregulatory axis remains unclear. Moreover, a role for Th17 cells cannot be excluded, however we were not able to detect IL-17 production by CD4^+^ T cells in the context of *Il27ra*-deficiency in this infection model.

TCR signalling increases the import of glucose and glutamine which promotes effector T cell differentiation [34,66,67]. Activated T cells preferentially use glycolysis for proliferation and to maintain effector function [34,66,67]. Additionally, mitochondrial ROS is critical for activation of NFAT and subsequent IL-2 production in T cells [45]. Consistent with these findings, we observed an increase in mitochondrial derived ROS in CD4^+^ T cells from *Il27ra*^*-/-*^ mice, indicative of their highly activated state, also supported by increased cell size and granularity. We hypothesised that IL-27 signalling limited glycolysis in CD4^+^ T cells as a means to prevent the hyper-activation, including excessive IFNγ and TNF production [68]. As previously reported, we also observed Th1 cells to be more glycolytic than their regulatory counterparts [48]. Recently, IL-27 and IL-15 signalling was shown to be critical for vaccine-elicited T cell responses, rather than infectious challenge [69]. CD8^+^ T cells responding to vaccination preferentially relied on mitochondrial function rather than aerobic glycolysis to support proliferation [69]. In support of these results we also show reduced IFNγ and TNF production by CD4^+^ T cells with 2-DG administration in a different infectious setting *in vivo*. Additionally, we noted decreased Th1 cell frequencies along with reduced IFNγ expression, suggesting IL-27 signalling limits glycolysis which is required for Th1 cell effector function [34].

The splenic architecture plays a crucial role in orchestrating and directing the trafficking of lymphocytes [70]. During VL, the breakdown of the splenic architecture is mediated by IFNγ and TNF [12,39], and comprises compartment-specific remodelling [71], as well as neovascularisation mediated by Ntkr2 [72]. Consistent with these findings [12], we showed glycolysis blockade in *Il27ra*^*-/-*^ mice rescued splenic architecture by preserving marginal zone macrophages, reducing IFNγ and TNF levels and retaining T and B cells in the spleen.

The breakdown of glucose into pyruvate during glycolysis provides metabolic intermediates that enter other metabolic pathways in order to contribute to amino acid synthesis and cell growth [73]. Our findings presented here, show that in the absence of IL-27 signalling, Th1 cells and their ability to produce IFNγ and TNF is impaired in the presence of 2-DG or heptelidic acid. Hence, inhibition of the first and middle steps of glycolysis results in reduced cytokine production by *bona fide* Th1 cells during infection and had no impact on Tr1 cells, suggesting that IL-27 signalling regulates glycolysis specifically in Th1 cells. Blocking glycolysis can reduce disease severity in inflammatory environments such as those found in arthritis [74,75], cancer [76], autoimmunity [77] and infections [48]. Our findings presented here highlight the importance of understanding the metabolic changes that occur during inflammation and how these pathways could potentially be targeted to restore functionality to tissues affected by inflammatory-mediated damage.

In conclusion, inflammation is a natural process that serves to protect the host against pathogens. Excess inflammation in the absence of appropriate immune regulation leads to a myriad of inflammatory diseases such as rheumatoid arthritis, colitis and multiple sclerosis. IL-27 signalling has been identified as a context-dependent modulator of inflammation in various settings. Here, we provide evidence for how IL-27 signalling limits Th1 cell-mediated host tissue damage by regulating glycolysis selectively in these cells. If left untreated, many inflammatory diseases progress to a chronic state resulting in dysfunctional immune responses and further host tissue damage. We show that targeting glycolysis provides an opportunity to dampen inflammation and rescues host tissue damage without compromising anti-parasitic immunity.

## Materials and methods

### Ethics statement

All animal procedures were approved by the QIMR Berghofer Medical Research Institute Animal Ethics Committee. This work was conducted under QIMR Berghofer animal ethics approval number A1707615M, in accordance with the “Australian Code of Practise for the Care and Use of Animals for Scientific Purposes” (Australian National Health and Medical Research Council).

### Mice

Female mice aged between 8–12 weeks were used for all experiments unless stated otherwise. C57BL/6J female mice aged 8–12 weeks were purchased from the Australian Resource Centre (Canning Vale, WA, Australia). All mice were maintained under pathogen-free conditions at the QIMR Berghofer Medical Research Institute Animal Facility (Herston, QLD, Australia). Mice were bred in-house including: *C57BL/6N* (C57BL/6N, RRID: IMSR_JAX:005304), B6N.129P2-*Il27ra*^*tm1Mak*^/J (*Il27ra*^*-/-*^, RRID: IMSR_JAX:018078)[78], B6.129S7-Rag1^tm1Mom^/J (*Rag1*^*-/-*^, RRID: IMSR_JAX:002216)[79], B6.129S6-*Il10*^*tm1Flv*^/J (*Il10* GFP, RRID: IMSR_JAX: 008379)[80], C57BL/6-*Foxp3*^*tm1Flv*^/J (*Foxp3*RFP, RRID: IMSR_JAX:008374)[81] and B6.129S4-*Ifng*^*tm3*.*1Lky*^/J (*Ifng*YFP, RRID: IMSR_JAX: 017581)[82].

### Parasites and infections

*Leishmania donovani* (clone LV9) [83] parasites were maintained by *in vivo* passage in *Rag1*^*-/-*^ mice and amastigotes were isolated from the spleens of chronically infected mice. Mice were infected with 2x10^7^ *L*. *donovani* amastigotes intravenously (i.v.) via the lateral tail vein. Spleen and liver impression smears were used to determine mean parasite burdens and expressed as Leishman Donovan Units (LDU = number of amastigotes per 1000 host nuclei multiplied by the organ weight in grams).

### *In vitro* infection

Peritoneal cells were collected from WT and *Il27ra*^*-/-*^ mice by peritoneal lavage and washed with complete DMEM (10% v/v FCS containing 10mM L-glutamine, 100U/mL penicillin and 100mg/ml streptomycin). 5x10^5^ cells were seeded in a 8-well glass chamber slide (NUN15491, Lab Tek, Thermo Fisher Scientific Australia Pty Ltd, Scoresby, VIC, Australia). After 24 hours, non-adherent cells were washed and removed with complete DMEM and 10 *L*. *donovani* amastigotes per cell (MOI = 10:1) was added and incubated for 1 hour at 37°C. After 1 hour, macrophages were washed and free amastigotes removed. Cells were cultured for another 24 hours with or without 25ng/mL of recombinant IFNγ (505812, BioLegend, California, USA) or 100ng/ml recombinant IL-27 (577404, BioLegend, California, USA). The following day, cells were washed with 1x dPBS, fixed in 95% (v/v) methanol and stained using 10% (v/v) Giemsa stain (GS500, Sigma Aldrich, NSW, Australia) diluted in water. The number of parasites or number of infected cells per 100 host macrophages was measured.

### *L*. *donovani* antigen re-stimulation assay

Spleens were harvested and processed through a 100μm cell strainer to obtain a single cell suspension. Splenocyte cell suspensions were then counted and adjusted to a concentration of 2x10^6^ cells/mL. *L*. *donovani* amastigotes (fixed in 4% PFA) were thawed and washed in RPMI media containing Penicillin-Streptomycin and then counted and adjusted to a final concentration of 4x10^7^/ml. Cells and parasites were plated into a 96-U bottom well plate at a 1:20 ratio, were each well contained 1x10^5^ cells and 2x10^6^ parasites. Cells were then cultured with the fixed *L*. *donovani* amastigotes for 24–72 hours. Culture supernatants were harvested at 24 and 72 hours post culture.

### Fluorescence microscopy

Mice were injected with 100μg i.v. of FITC dextran (D7137, Life Technologies Australia Pty Ltd, VIC, Australia), one day prior to collection of organs. Spleen tissue was collected into 4% (w/v) PFA (in PBS), incubated at room temperature for 1–2 hours and then transferred to a 30% (w/v) sucrose solution (in MilliQ water) overnight at 4°C. Fixed spleen tissue was then dabbed on filter paper to remove excess sucrose and subsequently snap frozen in O.C.T compound medium for cryotomy (00411243, Bio-strategy, Tingalpa, QLD, Australia). Splenic architecture and distribution of marginal zone macrophages (MZMs) were analysed in 20μm sections counter-stained with DAPI and visualised on the Aperio FL slide scanner. Image analysis was performed using Image Scope to determine area of the sections and Metamorph 7.8 (Integrated Morphometry analysis tool) (Molecular Devices, CA, USA) to count the number of MZMs per mm^2^.

### Cell processing for flow cytometry

Spleen and liver mononuclear cells were prepared for flow cytometric analysis by disassociating the tissue through a 100μm cell strainer or metal mesh. Single cell suspensions were then treated with RBC Lysis Buffer (R7757-100ML, Sigma Aldrich, NSW, Australia) for 7 minutes at room temperature to lyse red blood cells in each sample. Single cell suspensions were then centrifuged at 1300 rpm for 6 minutes and supernatant was decanted. Cell pellet was resuspended in 5ml of 1% FCS (in PBS) and cell viability was determined using trypan blue exclusion dye (C10228, Thermo Fisher Scientific Australia Pty Ltd, Scoresby, VIC, Australia). 2-5x10^6^ cells per well were plated in a 200μl volume in a 96 U-bottom well plate. Dead cells were excluded from the analysis using LIVE/DEAD fixable Aqua stain (423102, BioLegend, California, USA), as per manufacturer’s instructions. For detection of intracellular cytokines, cells were re-stimulated with 25ng/ml of PMA (P8139-1MG, Sigma Aldrich, NSW, Australia), 2μg/ml of Ionomycin (I0634-1MG, Sigma Aldrich, NSW, Australia) in the presence of 10μg/mL of Brefeldin A (B6542-5MG, Sigma Aldrich, NSW, Australia) for 3 hours at 37°C. Staining for cell surface markers was performed by incubating cells with cell surface antibodies for 30 minutes at 37°C and subsequently washing with FACS buffer. Intracellular cytokine staining was then performed by permeabilising the cells with the Foxp3 Transcription Buffer Staining kit (00-5523-00, Thermo Fisher Scientific Australia Pty Ltd, Scoresby, VIC, Australia), as per manufacturer’s instructions. Cells were then resuspended in 100μl of FACS buffer and acquired on a BD LSR Fortessa 5 (Special order research product, BD Biosciences, Macquarie Park, NSW, Australia).

### Flow cytometry

Mouse monoclonal antibodies: FITC-conjugated anti- CD11a (clone M17/4), PeCy7-conjugated anti-CD49d (clone R1-2), FITC or APC- conjugated anti-Foxp3 (clone MF-14), AF700 conjugated or BV785 conjugated anti-CD8a (clone 53–5.8), PerCP/Cy5.5 conjugated anti-CD11b (clone M1/70), PeCy7-conjugated anti-Ly6C (clone HK1.4), APC/Cy7 conjugated anti-Ly6G (clone 1A8), APC-conjugated anti-CD11c (clone N418), Pacific Blue-conjugated anti-MHCII (clone M5.114–15.3), BV650-conjugated anti-B220 (clone RA3-6B2), APC/Cy7 conjugated anti-NK1.1 (clone PK136), PE-conjugated anti- F4/80 (clone RMT4-54), PE/dazzle-conjugated anti- IL-10 (clone JES5-16E3), PeCy7 conjugated anti-Tbet (clone 4B10), APC-conjugated anti-IFNγ (clone XMG1.2), PE-conjugated anti-TNF (clone MP6-XT22) and PE-conjugated anti-IL-27p28 (clone MM27-7B1) were purchased from BioLegend (BioLegend, California, USA). BUV395-conjugated anti-CD4 (clone GK1.5) and BUV737-conjugated anti- TCRβ chain (clone H57-597) were purchased from BD Biosciences (BD Biosciences, NSW, Australia). Efluor660-conjugated anti-Tbet (clone ebio4B10) was purchased from eBioscience (Thermo Fisher Scientific Australia Pty Ltd, Scoresby, VIC, Australia). I-A^b^-PEPCK_335-351_ APC tetramer [38] was obtained from the NIH, Core tetramer facility (Emory University, Atlanta, GA, USA).

### Measurement of cytokine levels in the serum and/or cell culture supernatants

Cytokine levels in the serum and culture supernatants were measured using BD CBA, as per manufacturer’s instructions using the mouse inflammation kit (552364, BD Biosciences, Macquarie Park, NSW, Australia). Briefly, standards were prepared by serial dilutions (0-5000pg/mL) and subsequently the master mix containing antibody-coated beads along with the detection reagent (PE) was plated into a 96 V-bottom well-plate and standards and serum or supernatant samples were added and incubated in the master mix for 2 hours at room temperature. Samples were then washed with the 1x CBA washing buffer and resuspended in a final volume of 80μL of wash buffer before being acquired on the HTS system plate reader on the Fortessa 5 (Special order research product, BD Biosciences, Macquarie Park, NSW, Australia). Analysis was performed using the FCAP Array v3.0 software (Soft Flow, Minnesota, USA).

### Th1 cell *in vitro* polarisation

100μL of 5μg/mL Ultra-LEAF purified anti-mouse CD3ε (100340; clone 145-2C11; BioLegend, California, USA), diluted in 1x dPBS (Gibco) was added to each well (except media only controls) of a 96 U-well bottom plate and incubated for 2 hours in a 37°C incubator to coat the wells. Mouse T cell media was prepared under sterile conditions and filtered, 10% FBS (Gibco), 0.05mM β-mercaptoethanol (Sigma Aldrich, NSW, Australia), 1x non-essential amino acids (Gibco), 100U/mL penicillin and 100μg/mL streptomycin (penicillin-streptomycin, Gibco), in Dulbecco’s Modified Eagle Medium containing 4.5g/L D-Glucose, L-Glutamine and 1mM sodium pyruvate (Gibco). Media was allowed to reach 37°C prior to use. After coating, the purified anti-mouse CD3ε was discarded and 100μL of a 2x polarisation cocktail, prepared in mouse T cell media, was added to each respective well. Briefly, media only well contained, 100μL of mouse T cell media, as described above. Th0 conditions contained, 2μg/mL of Ultra-LEAF purified anti-mouse CD28 (102116; clone 37.51; BioLegend, California, USA) and 20ng/mL of recombinant mouse IL-2 (575404, BioLegend, California, USA). Th1 conditions contained, 2μg/mL of anti-mouse CD28 (102102; clone 37.51; BioLegend, California, USA), 20ng/mL of recombinant mouse IL-2 (575404, BioLegend, California, USA), 10μg/mL of anti-IL-4 (504122, clone 11B11, BioLegend, California, USA) and 10ng/mL of recombinant mouse IL-12p70 (577004, BioLegend, California, USA). After 72 hours, efficiency of polarisation was assessed by measuring Tbet, IFNγ, ΙL-10 and Foxp3 expression by flow cytometry (please see S2C Fig)

### Assessment of cellular metabolism

3-4x10^5^ CD4^+^ T cells were plated per well and immobilised by coating plates with 0.6μg Corning Cell Tak adhesive (354240, In Vitro Technologies Inc., QLD, Australia) or 1μg Poly-D-lysine (P6407, Sigma Aldrich, NSW, Australia) prior to running the Glycolysis stress test assay (SEA103020100, In Vitro Technologies Inc., QLD, Australia) as per manufacturer’s instructions. XF Base medium (SEA103335100, In Vitro Technologies Inc., QLD, Australia), supplemented with 1mM L-Glutamine (G7513, Sigma Aldrich, NSW, Australia) was prepared fresh on the day and pH adjusted to 7.4 at 37°C. For Fig 4G, 100ng/mL of recombinant mouse IL-10 (575806, BioLegend, California, USA) was added to port A, glucose to port B, oligomycin to port C and 2-DG to port D.

### MitoTracker staining

200nM of MitoTracker Green (M7514), 200nM MitoTracker Deep Red (M22426) (Life Technologies Australia Pty Ltd, VIC, Australia) and 5μM of MitoSOX Red (M36008) (Life Technologies Australia Pty Ltd, VIC, Australia) were used to identify mitochondria and mROS by flow cytometry. Cells were incubated with MitoTracker dyes for 30 mins at 37°C and then washed with HBSS (14175–103, Life Technologies Australia Pty Ltd, VIC, Australia) and then acquired on a BD FSR Fortessa 5 (BD Biosciences; special order research product).

### *In vivo* 2-DG administration

Mice were injected with either PBS (controls) or 2-DG i.p. (D6134, Sigma Aldrich, NSW, Australia) at a dose of 1g/kg every for 7 days, beginning at day 7 p.i. until day 14 p.i.

### *In vitro* glycolysis blockade

Spleens and livers were harvested from WT and *Il27ra*^*-/-*^ mice 14 days p.i. and CD4^+^ T cells were MACS purified as above. 2x10^5^ CD4^+^ T cells were plated out in combination with media or 1mM of either 2-DG (D6134, Sigma Aldrich, NSW, Australia) or heptelidic acid (14079, Sapphire Bioscience Pty Ltd, NSW, Australia) and incubated at 37°C for 1 hour and then re-stimulated with 25ng/ml of PMA (P8139-1MG, Sigma Aldrich, NSW, Australia), 2μg/ml of Ionomycin (I0634-1MG, Sigma Aldrich, NSW, Australia) in the presence of 2μM of Monensin (420701, BioLegend, California, USA) for 3 hours at 37°C. CD4^+^ T cells were then stained for cell surface markers and intracellular markers, as described above and acquired on a BD LSR Fortessa 5 (BD Biosciences; special order research product).

### Liver granulomas

7μm frozen sections were dried overnight then fixed in a 3:1 mix of anhydrous acetone/ethanol for 5 minutes. Endogenous peroxidase was blocked using 0.5% (v/v) hydrogen peroxide in methanol for 10 minutes and non-specific binding of the primary antibody minimized by incubating sections in Biocare Medical Background Sniper plus 2.0% (w/v) BSA for 10 minutes. The primary antibody, hamster anti-mouse LV9 diluted 1:1000 in Biocare Medical Da Vinci Green antibody diluent, was applied for 60 minutes then detected by applying Jackson Immunoresearch goat anti-hamster secondary, diluted 1:300 in TBS, for 30 minutes followed by a 30 minute application of Vector ImmPRESS Goat HRP polymer. LV9 signal was visualized using Vector ImmPACT DAB. Sections were counterstained with Mayer’s Haematoxylin in a Leica Autostainer XL and mounted using a Leica CV5030 coverslipper.

### Granuloma image analysis

Granulomas were imaged in Image Processing and Analysis in Java [84] (Image J, NIH, Bethesda, Maryland, USA). Images were processed with the following settings: image threshold: 155–190, details of the script modified under: Granuloma_macro_edit2.

### Statistical analysis

Statistical analysis was performed using GraphPad Prism software version 7.02 (Graphpad, San Diego, CA, USA). Two independent groups were compared using a non-parametric Mann-Whitney U test, where *p<0.05, **p<0.01 was considered to be statistically significant. Groups with two or more dependent variables were compared using a One-Way ANOVA with Tukey’s multiple comparisons test or a Two-Way ANOVA with Sidak’s multiple comparisons test, where *p<0.05, **p<0.005, ***p<0.001, **** p<0.0001. All data are presented as the mean ± SEM.

## Supporting information

S1 Fig IL-27 signalling regulates CD4^+^ T cell responses during *L*. *donovani* infection. C57BL/6J mice were infected with 2x10^7^ *L*. *donovani* amastigotes i.v.. Antigen (PEPCK^+^)-specific CD4^+^ T cell responses measured by flow cytometry in the (**A**) spleen and (**B**) liver 14 days p.i. Antigen-specific CD4^+^ T cells defined as Foxp3^-^CD4^+^ TCRβ^+^ CD44^+^ PEPCK^+^. From the antigen-specific CD4^+^ T cell gate, Tr1 cells defined as IL-10^+^ IFNγ^+^ and from the IFNγ^+^ IL-10^-^ gate, Th1 cells defined as Tbet^+^ IFNγ^+^. Line graphs track the frequencies of antigen-specific CD4^+^ T cells throughout the course of infection including, day 0, 7, 14, 28 and 56 p.i. in the spleen (white circles) and liver (black circles). (**C**) WT and *Il27ra*^*-/-*^ mice were infected with 2x10^7^ *L*. *donovani* amastigotes i.v.. CD4^+^ T cell frequencies defined as CD4^+^ TCRβ^+^. Antigen specific CD4^+^ T cell frequencies defined as CD44^+^ PEPCK^+^. Antigen specific Th1 cell frequencies defined as CD44^+^ PEPCK^+^ Tbet^+^ IFNγ^+^. Antigen specific Tr1 cell frequencies defined as CD44^+^ PEPCK^+^ IL-10^+^ IFNγ^+^. Antigen specific Treg cell frequencies defined as CD44^+^ PEPCK^+^ Foxp3^+^ IL-10^+^by flow cytometry in the spleen and liver. (**D**) IFNγ, TNF and IL-10 levels (pg/mL) measured in the serum 14 days p.i. (**E**) WT and *Il27ra*^*-/-*^ mice were infected with 2x10^7^ *L*. *donovani* amastigotes i.v. and 14 days p.i. mitochondrial volume (Vol) and membrane potential (MP) was measured on Th1 cells identified as CXCR3^+^ CXCR5^-^ (gated on CD4^+^ TCRβ^+^) by flow cytometry. (**F**) WT and *Il27ra*^*-/-*^ mice were infected with 2x10^7^ *L*. *donovani* amastigotes i.v.. Th1 cell frequencies measured by flow cytometry in uninfected and infected mice *ex vivo* 14 days p.i. (**G**) Peritoneal cells were isolated from WT and *Il27ra*^*-/-*^ mice and incubated with *L*. *donovani* amastigotes for 24 hours with or without IFNγ or IL-27. Number of parasites or infected cells per 100 host macrophages are shown as a measure of infectivity. Data shown is representative of 2 independent experiments performed with n = 4–6 mice per group, in each experiment and are presented as mean ± SEM. C, D: **p<0.01, Mann-Whitney U test, F, G: ****p<0.0001, ***p<0.0005, **p<0.005, One-Way ANOVA with Tukey’s multiple comparisons test.
(TIF)

S2 Fig IL-27 signalling limits glycolysis to regulate cytokine production. WT and *Il27ra*^*-/-*^ mice were infected with 2x10^7^ *L*. *donovani* amastigotes i.v.. Splenic and hepatic CD4^+^ T cells were MACS purified and assayed on the Seahorse XF96 using the glycolysis stress test kit at day 14 p.i.. Total oxygen consumption rate (OCR) was assessed after the addition of glucose, oligomycin and 2-DG at indicated times in the (**A**) spleen and (**B**) liver. (**C**) Naïve WT splenic CD4^+^ T cells MACS purified and polarised to Th0 and Th1 conditions. 72 hours later polarisation efficiency assessed by measuring Tbet, IFNγ, IL-10 and Foxp3 expression by flow cytometry. (**D**) Splenic CD4^+^ T cells MACS purified from day 14 infected WT and *Il27ra*^*-/-*^ mice and treated with 100ng/mL of recombinant mouse IL-10 as part of the injection protocol, 30 minutes before the addition of glucose, oligomycin and 2-DG on the Seahorse XF96. Glycolytic capacity was calculated as: (Maximum rate measurement after Oligomycin injection)–(Last rate measurement before Glucose injection), Glycolysis (ECAR) measured in all conditions. Data shown is representative of 2 independent experiments performed with n = 5–6 mice per group, in each experiment and are presented as mean ± SEM, ***p<0.0005, One-Way ANOVA with Tukey’s multiple comparisons test.
(TIF)

S3 Fig 2-DG treatment exhibits a minor effect on immune cell populations in the spleen and CD4^+^ T cells are the predominant source of IFNγ. WT and *Il27ra*^*-/-*^ mice infected with 2x10^7^ *L*. *donovani* amastigotes i.v.. Mice were treated with PBS (controls) or 1g/kg of 2-DG daily i.p. beginning at day 7 p.i. until day 14 p.i. Organs were harvested 14 days p.i. and processed for cellular analysis. (**A**) Conventional and antigen-specific Tregs producing IL-10 were measured by flow cytometry 14 days p.i. (**B**) Gating strategy for CD4^+^ T cells (red), CD8^+^ T cells (blue), NK1.1^+^ (green), CD19^+^ (orange), CD11c^+^ (yellow), CD11b^+^ (purple), Ly6C^+^ (teal), Ly6G^+^ (pink), F4/80^+^ dextran^+^(grey) cells were analysed by flow cytometry in the spleen 14 days p.i. (**C**) Frequencies and numbers of CD4^+^ T cells, CD8^+^ T cells, NK1.1^+^, CD19^+^, CD11c^+^, CD11b^+^, Ly6C^+^, Ly6G^+^, F4/80^+^ dextran^+^ cells were analysed by flow cytometry in the spleen 14 days p.i. (**D**) Cellular sources of IFNγ was measured by flow cytometry in the spleen 14 days p.i. same gating strategy as described in **B**, but gating on total IFNγ^+^ events after the live/dead gate. Data shown is representative of 3 independent experiments performed with n = 4–6 mice per group, in each experiment and are presented as mean ± SEM, **p<0.005, One-Way ANOVA with Tukey’s multiple comparisons test.
(TIF)

S4 Fig IL-27 signalling limits glycolysis to regulate cytokine production by Th1 cells and not Tr1 cells. (**A**) 2x10^5^ CD4^+^ T cells were MACS purified from the spleens and livers of WT and *Il27ra*^*-/-*^ mice at day 14 p.i. and treated with either media, or 1mM of either 2-DG or heptelidic acid (HA) for 1 hour and re-stimulated with PMA/Ionomycin in the presence of monensin for 3 hours. Th1 (Tbet^+^ IFNγ^+^) cell frequencies were measured by flow cytometry. Plots for Tbet (x-axis) and IFNγ (y-axis) are shown for each treatment. (**B**) Tr1 (IL-10^+^ IFNγ^+^) cell frequencies shown in response to media, 2-DG and HA treatment, as described in S4A Fig. Data shown is representative of 2 independent experiments performed with n = 5 mice per group, in each experiment and are presented as mean ± SEM, *p<0.05, Two-Way ANOVA with Sidak’s multiple comparisons test.
(TIF)
