# Supplementary figures and images for: IL-27 signalling regulates glycolysis in Th1 cells to limit immunopathology during infection
Source: PLoS Pathog. 2020 Oct 13;16(10):e1008994. doi: 10.1371/journal.ppat.1008994 (PMC7584222; doi:10.1371/journal.ppat.1008994)

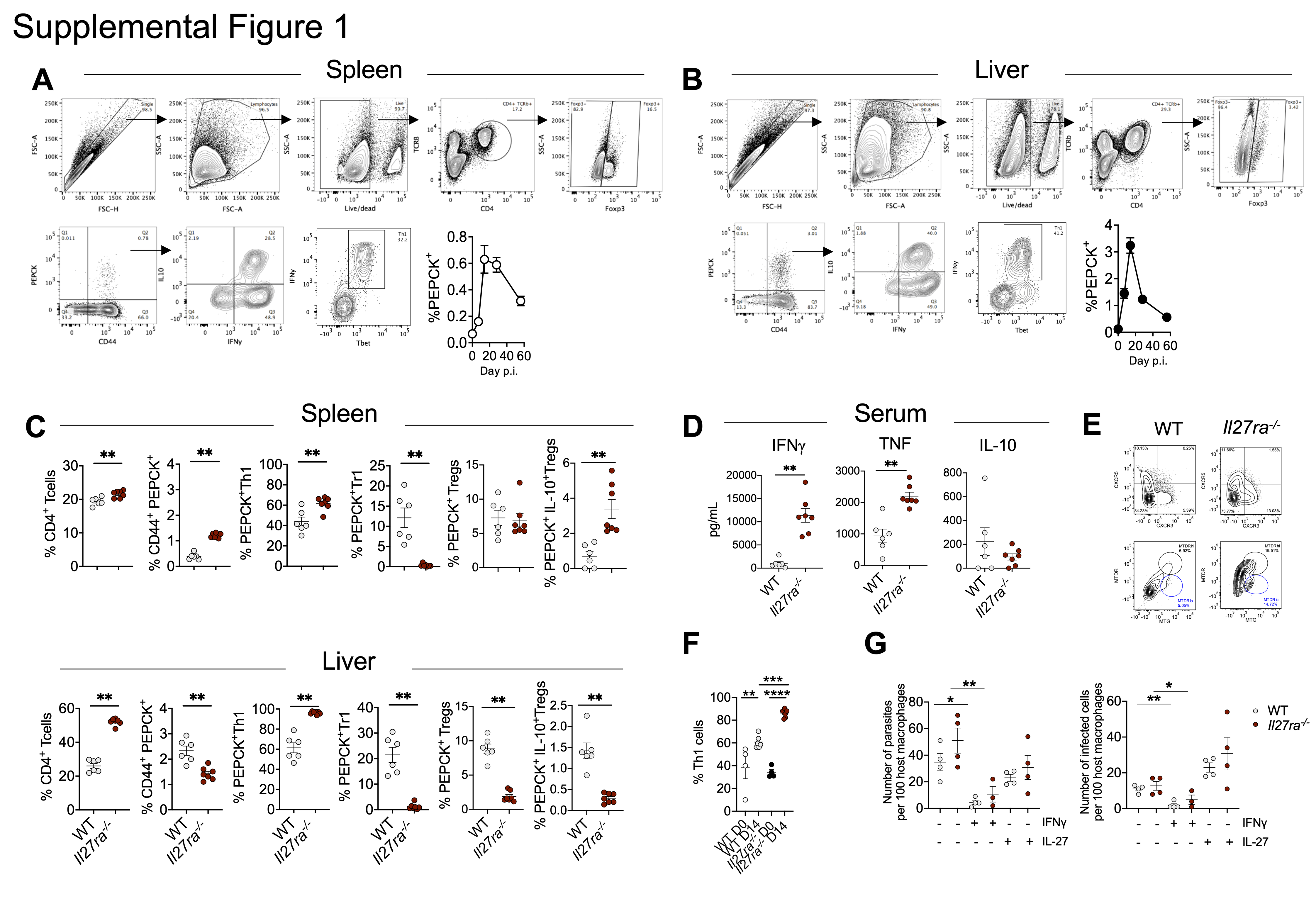

Supplement: S1 Fig — C57BL/6J mice were infected with 2x10^7^ L. donovani amastigotes i.v.. Antigen (PEPCK+)-specific CD4+ T cell responses measured by flow cytometry in the (A) spleen and (B) liver 14 days p.i. Antigen-specific CD4+ T cells defined as Foxp3-CD4+ TCRβ+ CD44+ PEPCK+. From the antigen-specific CD4+ T cell gate, Tr1 cells defined as IL-10+ IFNγ+ and from the IFNγ+ IL-10- gate, Th1 cells defined as Tbet+ IFNγ+. Line graphs track the frequencies of antigen-specific CD4+ T cells throughout the course of infection including, day 0, 7, 14, 28 and 56 p.i. in the spleen (white circles) and liver (black circles). (C) WT and Il27ra-/- mice were infected with 2x10^7^ L. donovani amastigotes i.v.. CD4+ T cell frequencies defined as CD4+ TCRβ+. Antigen specific CD4+ T cell frequencies defined as CD44+ PEPCK+. Antigen specific Th1 cell frequencies defined as CD44+ PEPCK+ Tbet+ IFNγ+. Antigen specific Tr1 cell frequencies defined as CD44+ PEPCK+ IL-10+ IFNγ+. Antigen specific Treg cell frequencies defined as CD44+ PEPCK+ Foxp3+ IL-10+by flow cytometry in the spleen and liver. (D) IFNγ, TNF and IL-10 levels (pg/mL) measured in the serum 14 days p.i. (E) WT and Il27ra-/- mice were infected with 2x10^7^ L. donovani amastigotes i.v. and 14 days p.i. mitochondrial volume (Vol) and membrane potential (MP) was measured on Th1 cells identified as CXCR3+ CXCR5- (gated on CD4+ TCRβ+) by flow cytometry. (F) WT and Il27ra-/- mice were infected with 2x10^7^ L. donovani amastigotes i.v.. Th1 cell frequencies measured by flow cytometry in uninfected and infected mice ex vivo 14 days p.i. (G) Peritoneal cells were isolated from WT and Il27ra-/- mice and incubated with L. donovani amastigotes for 24 hours with or without IFNγ or IL-27. Number of parasites or infected cells per 100 host macrophages are shown as a measure of infectivity. Data shown is representative of 2 independent experiments performed with n = 4–6 mice per group, in each experiment and are presented as mean ± SEM. C, D: **p<0.01, Mann-Whitney [file ppat.1008994.s001.tif]

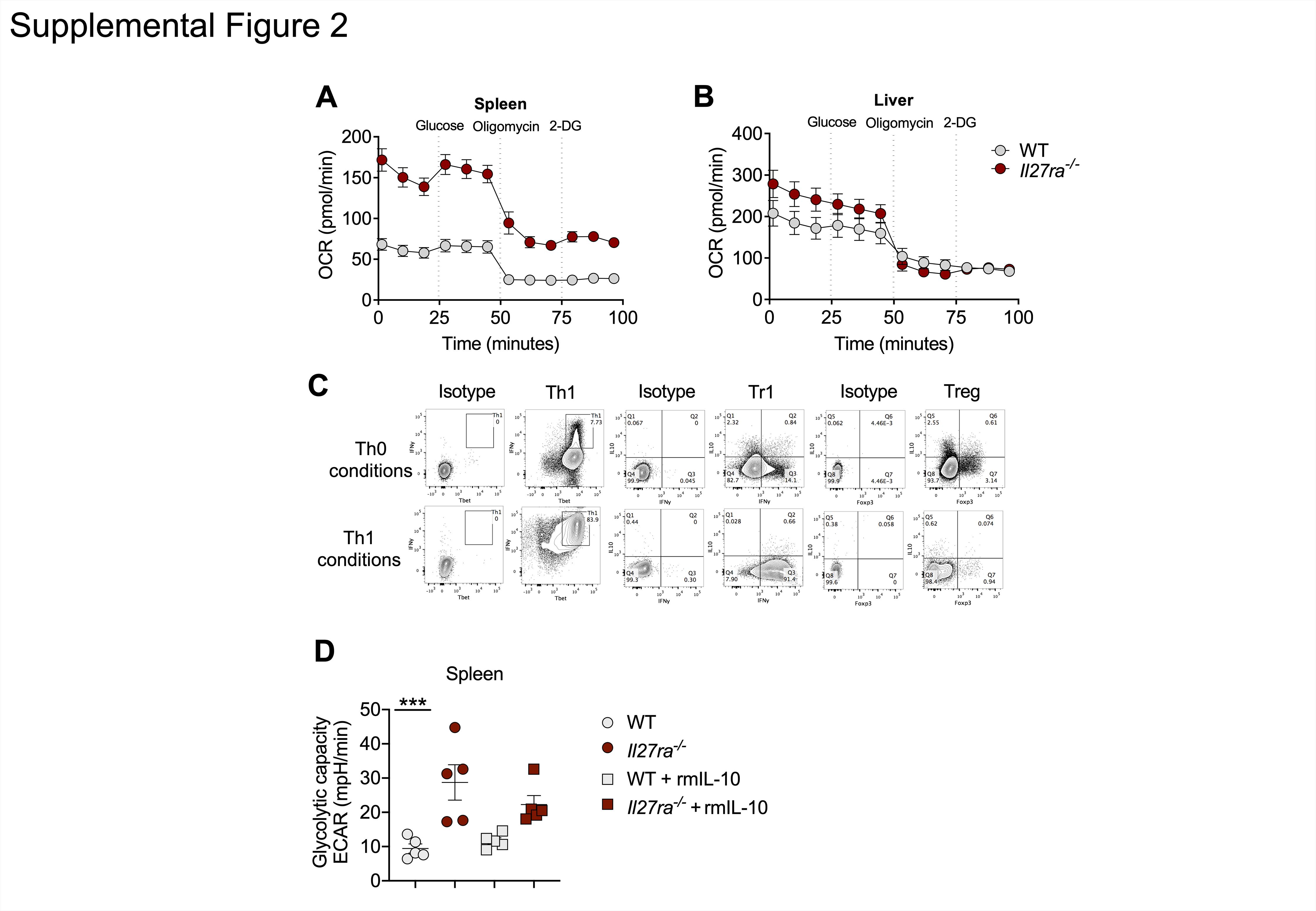

Supplement: S2 Fig — WT and Il27ra-/- mice were infected with 2x10^7^ L. donovani amastigotes i.v.. Splenic and hepatic CD4+ T cells were MACS purified and assayed on the Seahorse XF96 using the glycolysis stress test kit at day 14 p.i.. Total oxygen consumption rate (OCR) was assessed after the addition of glucose, oligomycin and 2-DG at indicated times in the (A) spleen and (B) liver. (C) Naïve WT splenic CD4+ T cells MACS purified and polarised to Th0 and Th1 conditions. 72 hours later polarisation efficiency assessed by measuring Tbet, IFNγ, IL-10 and Foxp3 expression by flow cytometry. (D) Splenic CD4+ T cells MACS purified from day 14 infected WT and Il27ra-/- mice and treated with 100ng/mL of recombinant mouse IL-10 as part of the injection protocol, 30 minutes before the addition of glucose, oligomycin and 2-DG on the Seahorse XF96. Glycolytic capacity was calculated as: (Maximum rate measurement after Oligomycin injection)–(Last rate measurement before Glucose injection), Glycolysis (ECAR) measured in all conditions. Data shown is representative of 2 independent experiments performed with n = 5–6 mice per group, in each experiment and are presented as mean ± SEM, ***p<0.0005, One-Way ANOVA with Tukey’s multiple comparisons test. (TIF) [file ppat.1008994.s002.tif]

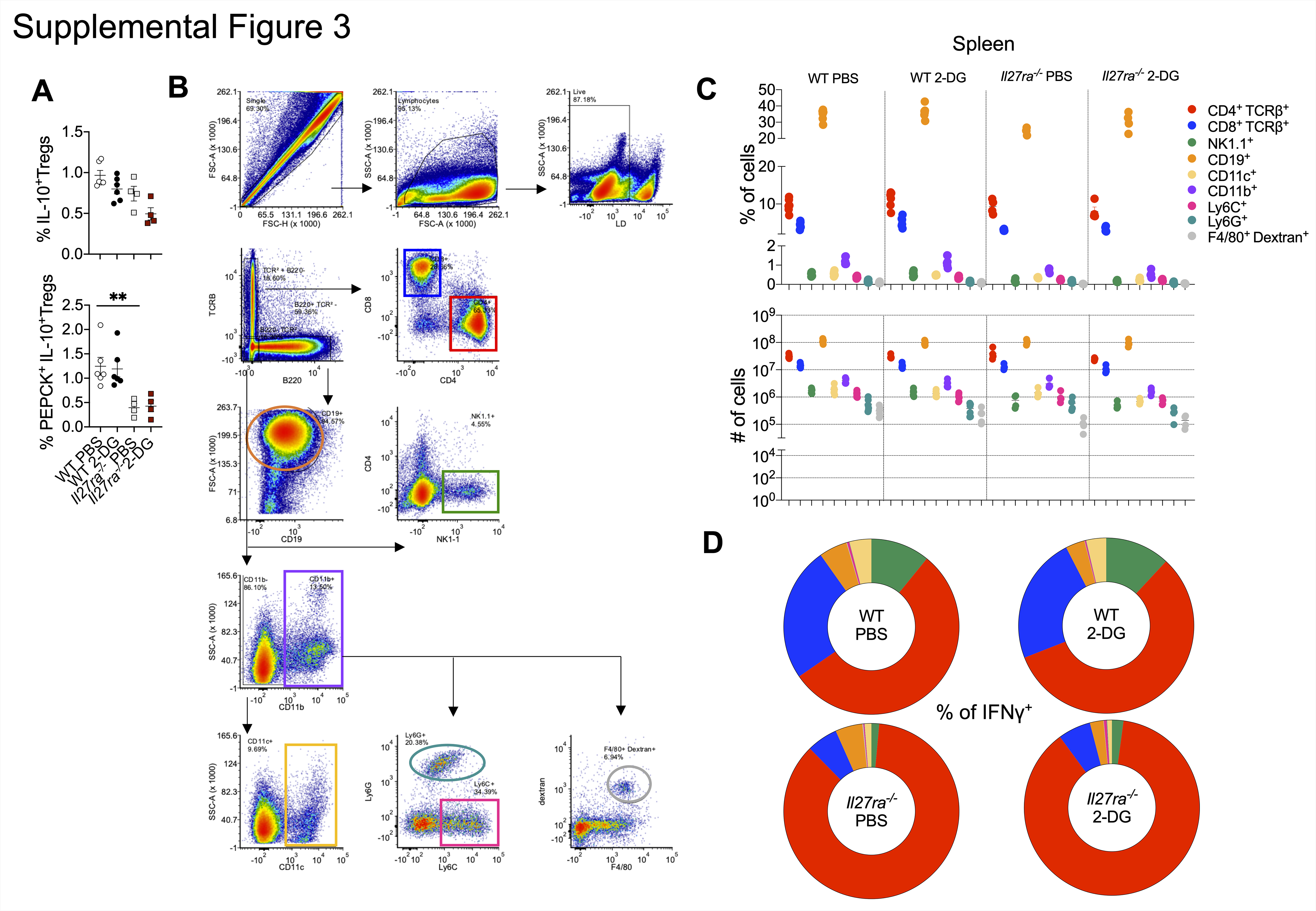

Supplement: S3 Fig — WT and Il27ra-/- mice infected with 2x10^7^ L. donovani amastigotes i.v.. Mice were treated with PBS (controls) or 1g/kg of 2-DG daily i.p. beginning at day 7 p.i. until day 14 p.i. Organs were harvested 14 days p.i. and processed for cellular analysis. (A) Conventional and antigen-specific Tregs producing IL-10 were measured by flow cytometry 14 days p.i. (B) Gating strategy for CD4+ T cells (red), CD8+ T cells (blue), NK1.1+ (green), CD19+ (orange), CD11c+ (yellow), CD11b+ (purple), Ly6C+ (teal), Ly6G+ (pink), F4/80+ dextran+(grey) cells were analysed by flow cytometry in the spleen 14 days p.i. (C) Frequencies and numbers of CD4+ T cells, CD8+ T cells, NK1.1+, CD19+, CD11c+, CD11b+, Ly6C+, Ly6G+, F4/80+ dextran+ cells were analysed by flow cytometry in the spleen 14 days p.i. (D) Cellular sources of IFNγ was measured by flow cytometry in the spleen 14 days p.i. same gating strategy as described in B, but gating on total IFNγ+ events after the live/dead gate. Data shown is representative of 3 independent experiments performed with n = 4–6 mice per group, in each experiment and are presented as mean ± SEM, **p<0.005, One-Way ANOVA with Tukey’s multiple comparisons test. (TIF) [file ppat.1008994.s003.tif]

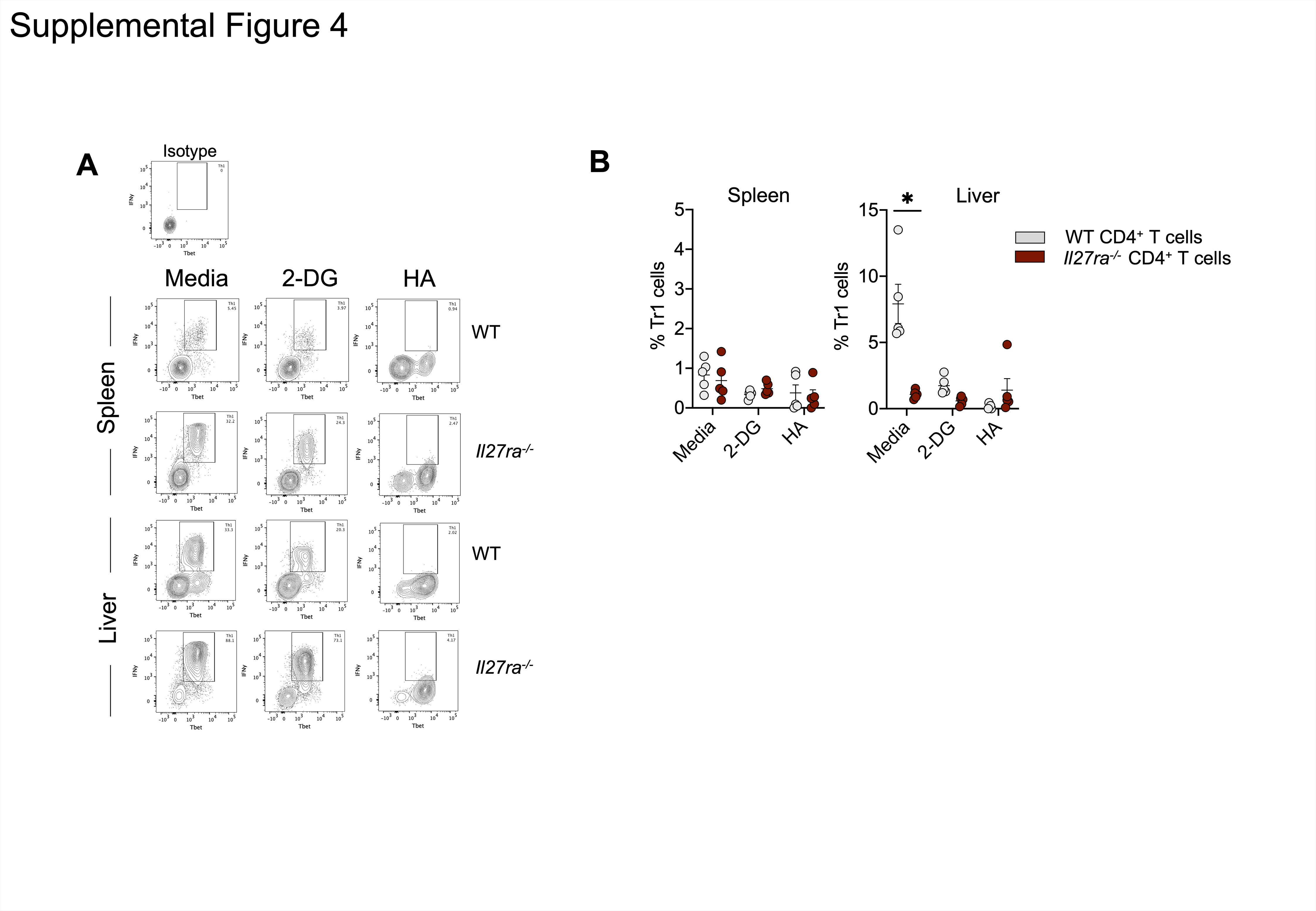

Supplement: S4 Fig — (A) 2x10^5^ CD4+ T cells were MACS purified from the spleens and livers of WT and Il27ra-/- mice at day 14 p.i. and treated with either media, or 1mM of either 2-DG or heptelidic acid (HA) for 1 hour and re-stimulated with PMA/Ionomycin in the presence of monensin for 3 hours. Th1 (Tbet+ IFNγ+) cell frequencies were measured by flow cytometry. Plots for Tbet (x-axis) and IFNγ (y-axis) are shown for each treatment. (B) Tr1 (IL-10+ IFNγ+) cell frequencies shown in response to media, 2-DG and HA treatment, as described in S4A Fig. Data shown is representative of 2 independent experiments performed with n = 5 mice per group, in each experiment and are presented as mean ± SEM, *p<0.05, Two-Way ANOVA with Sidak’s multiple comparisons test. (TIF) [file ppat.1008994.s004.tif]
